# Herceptin-Mediated Cardiotoxicity: Assessment by Cardiovascular Magnetic Resonance

**DOI:** 10.1155/2022/1910841

**Published:** 2022-02-27

**Authors:** Jin Jiang, Boyang Liu, Sandeep S Hothi

**Affiliations:** ^1^Heart and Lung Centre, New Cross Hospital, Wolverhampton, UK; ^2^Institute of Cardiovascular Sciences, College of Medical and Dental Sciences, University of Birmingham, Birmingham, UK

## Abstract

Herceptin (trastuzumab) is a recombinant, humanized, monoclonal antibody that targets the human epidermal growth factor receptor 2 (HER2) and is used in the treatment of HER2-positive breast and gastric cancers. However, it carries a risk of cardiotoxicity, manifesting as left ventricular (LV) systolic dysfunction, conventionally assessed for by transthoracic echocardiography. Clinical surveillance of cardiac function and discontinuation of trastuzumab at an early stage of LV systolic dysfunction allow for the timely initiation of heart failure drug therapies that can result in the rapid recovery of cardiac function in most patients. Often considered the reference standard for the noninvasive assessment of cardiac volume and function, cardiac magnetic resonance (CMR) imaging has superior reproducibility and accuracy compared to other noninvasive imaging modalities. However, due to limited availability, it is not routinely used in the serial assessment of cardiac function in patients receiving trastuzumab. In this article, we review the diagnostic and prognostic role of CMR in trastuzumab-mediated cardiotoxicity.

## 1. Introduction

Herceptin (trastuzumab) is a recombinant, humanized, monoclonal antibody directed against the extracellular domain IV of the human epidermal growth factor receptor 2 (HER2) and is indicated for the treatment of HER2-positive breast and gastric cancers [1–3]. HER2 positivity is relatively frequent, found in around one-fifth of breast and gastric cancer patients [4, 5]. Trastuzumab has been transformational for the prognosis of these patients, acting through its mechanisms of preventing HER2 dimerization and downstream signalling, HER2 internalization and degradation, and antibody-dependent cellular cytotoxicity [6, 7].

Although the chemotherapeutic mechanisms of trastuzumab are well characterised, the molecular aspects of trastuzumab-induced cardiotoxicity, recognised since its phase III trial [8], remain incompletely understood. Early studies have reported trastuzumab-related cardiotoxicity to be largely reversible with endomyocardial biopsies demonstrating an absence of the typical anthracycline-induced cardiomyocyte vacuolization or dropout [9]. However, in vivo mice studies have found trastuzumab to alter the expression of 15 genes involved in cardiac contractility, adaptation to stress, as well as DNA repair, cellular proliferation, healing, and mitochondrial function [10]. Furthermore, trastuzumab-mediated phosphorylation of HER1 and HER2 has been reported to activate the autophagy inhibitory Erk signalling pathway in human primary cardiomyocytes, inducing cardiotoxicity by disrupting the cardiomyocyte's ability to recycle cellular toxins [11]. These data, together with analyses of major trastuzumab trials, have highlighted the potential for trastuzumab to induce persistent left ventricular (LV) systolic dysfunction (LVSD) despite drug cessation [12]. This is of concern particularly as heart failure induced by cancer therapy is associated with worse outcomes than that of more common heart failure patients [13]. Despite this, it is important to recognise that close clinical surveillance and discontinuation of trastuzumab at an early stage of LVSD will allow the timely initiation of heart failure drug therapies that can result in the rapid recovery of cardiac function in most patients [1, 14].

Consequently, a distinct multidisciplinary clinical subspecialty, cardio-oncology, has emerged with the aim of preventing, monitoring, and treating cancer therapeutics-related cardiac dysfunction (CTRCD) [15]. In current cardio-oncology practice, transthoracic echocardiography (TTE) remains the first line for cardiac surveillance among oncology patients due to its widespread availability and lack of radiation exposure [16–19]. However, with a reported temporal inter- and intra-observer variability of 10% in the assessment of left ventricular ejection fraction (LVEF) by 2D TTE [20], cardiac magnetic resonance (CMR) is gaining an increasingly prominent role in cardio-oncology. Often considered the reference standard for the assessment of cardiac volume and function, CMR has demonstrated superior reproducibility and accuracy compared to other conventional methods [21]. However, due to limited availability, it is not widely used in the serial monitoring for cardio-oncology assessment. Here in this article, we aim to review the diagnostic and prognostic role of CMR in trastuzumab-mediated cardiotoxicity.

## 2. Volumetric Assessment and CMR

**Figure 1:**
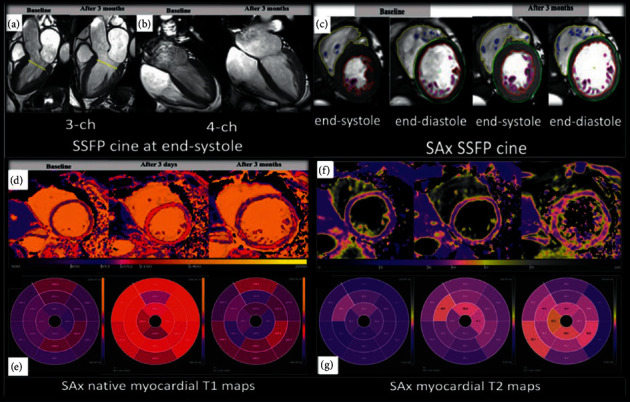
Representative images of trastuzumab-induced cardiotoxicity. Cine 3-chamber (a) and 4-chamber views (b) as well as short axis view (c) during baseline study (LVEF = 66%) and 3 months thereafter (LVEF = 54%). Native T1 (d) shows reduction of global left ventricular value after 3 days (baseline = 1196 ms, 1st follow-up = 1172 ms, and 2nd follow-up = 1277 ms). Myocardial T2 (f) shows subtle elevation of global value after 3 months (baseline = 40 ms, 1st follow-up = 43 ms, and 2nd follow-up = 44 ms). The average segmental T1 and T2 times are displayed as “bull's eye” images (e, g). The colour maps represent continuous T1 and T2 values. LVEF, left ventricular ejection fraction; reproduced with permission from Abdelmonem Atia et al. [22].

The assessment of cardiac function before, during, and after therapy is essential for all cancer patients undergoing potentially cardiotoxic therapy [16]. Whilst CMR is widely considered as the reference standard for cardiac volumetric assessment, its current role remains reserved for patients with inadequate echocardiographic windows due to limitations in availability, higher cost, and the requirement of patient cooperation with breath-holding and an absence of claustrophobia [1, 16, 21]. Conversely, echocardiography with its wider availability and cost-effectiveness is highly suited for serial surveillance. Consequently, given that definitions of cardiotoxicity in many oncology trials are based on a reduction of LVEF, TTE-derived LVEF remains the first-line method for the detection of CTRCD according to consensus guidelines [1, 16–19] (Figure 1). One of the key limitations of 2D TTE is its significant inter- and intra-observer variation, often quoted at 10% [16, 20]. Therefore, it can be challenging to discern whether a change in LVEF, for instance from 55 to 45%, represents true dysfunction or merely inter-study variation. This variability can be improved with the use of LV opacification contrast and is better still with 3D TTE [20, 23]. Similarly, 3D TTE has been reported to possess superior sensitivity (53%) to 2D TTE (25–29%) for the identification of LVEF <50% in adult survivors of childhood cancer when using CMR quantification as the reference standard [24]. However, it is evident from a recent survey of 96 echocardiographic laboratories from 22 different countries across Europe that there are wide variations in the adoption of 3D TTE with only 32% of centres routinely capturing 3D data for all TTE studies, and 20% of centres not performing any 3D TTE [25]. Furthermore, the feasibility of 3D TTE can be suboptimal even under research conditions. In a study of 100 breast cancer patients undergoing baseline and surveillance TTE during chemotherapy, 3D TTE was reported to be feasible in only 66% of studies, with factors such as increasing age, weight, smoking, mastectomy, and concomitant radiotherapy contributing to poor 3D image quality [26].

Multigated acquisition (MUGA) scanning was once a commonly used method for serial evaluation of cardiotoxicity. Despite low inter- and intra-observer variability, such methodology may be rendered obsolete in modern times due to low sensitivity to subtle changes and radiation exposure [27] (Table 1).

The superior accuracy and lower variability of CMR lend it clinical significance not only for the timely diagnosis of CTRCD, via detection of true positives cases, but also for its ability to avoid false negatives, thereby preventing unnecessary treatment interruptions. This is evident from a retrospective cohort study of 369 patients receiving trastuzumab therapy for breast cancer where trastuzumab was withheld for at least 4 weeks in patients who had experienced a decline in LVEF ≥16%, or decline ≥10% whilst below normal LVEF limits [28]. This treatment interruption allowed time for cardiology review and cardioprotective therapy initiation. Despite trastuzumab being recommenced in those whose LVEF recovered to normal, patients experiencing any treatment interruption possessed significantly worse outcomes in terms of both disease-free survival (adjusted hazards ratio of 4.4, *P*=0.001) and overall survival (adjusted hazards ratio 4.8, *P* < 0.001) [28].

In the absence of randomized prospective studies directly comparing patient outcomes from CMR and TTE derived LVEF, guidance from the British Society for Echocardiography (BSE) and British Cardio-Oncology Society (BCOS) recognises the addition of recent pilot data on the safe use of trastuzumab in patients with asymptomatic reductions in TTE-derived LVEF down to 40% [17, 29, 30]. These guidelines may help to compensate for the variability associated with TTE derived LVEF discussed above, emphasising the ESC's personalized approach to cardiac surveillance by cardio-oncology services [16], and support echocardiography in remaining at the core of cardio-oncology diagnostics.

## 3. Myocardial Strain and CMR

While LVEF has historically been used as a standard measure of systolic function, there is increasing interest in the use of more sensitive markers that can detect “subclinical” signs of LV dysfunction that can aid earlier initiation of cardioprotective therapy. The extent of myocardial deformation which occurs following application of contractile and relaxation forces can be quantified as strain, defined as the percent change in myocardial length from the relaxed to the contractile state. This deformation represents a fundamental property of the tissue [31] and there is increasing evidence for a causative relationship between the development of myocardial fibrosis and a reduction in ventricular deformation across a range of conditions [32–34]. Deformation imaging may, therefore, act as a functional imaging biomarker of myocardial fibrosis and offer additional prognostic information for the personalized management of patients receiving trastuzumab. Unlike the inherent flaws of a simplistic measurement such as LVEF, strain allows quantification of the different spatial components of contractile function in either longitudinal (GLS), circumferential (GCS), or radial (GRS) directions, both globally and regionally.

Most myocardial strain studies in patients receiving trastuzumab have used GLS derived from 2D speckle tracking echocardiography (STE). A meta-analysis of 9 studies found reduced GLS to be associated with a higher CTRCD risk (odds ratio 12.27; 95% CI 5.84–42.85; area under the hierarchical summary receiver operating characteristic curves 0.86; 95% CI, 0.83–0.89) [35]. However, there remains uncertainty regarding whether a strain-guided management approach offers incremental prognostic value compared to an LVEF-guided approach. In an observational study where 24 out of 81 consecutive women receiving trastuzumab developed CTRCD, GLS reduction was the strongest predictor of cardiotoxicity [36]. However, in the only prospective randomized controlled trial where 331 anthracycline-treated patients were randomized to either LVEF or GLS guided therapy, there were no significant differences in the primary outcome of change in LVEF between the two different study arms [37]. Despite this, it is important to recognise that the GLS-guided approach led to greater use of cardioprotective therapy, higher final LVEF, and lower incidence of CTRCD [37]. The current limitation of STE derived GLS is in its significant inter-vendor variability [38] with guideline quoted normal GLS values of <−17% for men and <−18% for women being specific to General Electric (United States) analysis software [17] alongside the demands for good image quality. Strain analysis of 3D STE datasets is also feasible. However, as a relatively novel technique, there is a lack of data for its use in CTRCD and it generally demands patients to breath-hold as well as a regular cardiac rhythm to enable multi-beat 3D acquisition [39].

Myocardial strain quantification is also feasible with CMR and is traditionally performed with one of many dedicated “tagging” sequences (such as spatial modulation of magnetization (SPAMM), harmonic phase (HARP), displacement encoding (DENSE), and strain encoding (SENC). These sequences magnetise temporary tags into the myocardium which are prominent during systole and fade during diastole. These tags can be tracked throughout the cardiac cycle to highlight myocardial movement. CMR-tagging derived GLS and GCS have been noted to be worse (less negative) than STE derived strain in a study of 46 cancer survivors exposed to anthracycline therapy with normal range LVEF, suggesting CMR to be more sensitive to subclinical LV dysfunction compared to TTE [40]. Looking beyond cardio-oncology, CMR-tagging GCS was again found to offer incremental predictive value to the traditional parameters of LVEF, left ventricular mass, and cardiovascular risk factors, for the future onset of heart failure in 1768 asymptomatic individuals from the Multi-Ethnic Study of Atherosclerosis (MESA) cohort [41].

The main disadvantage of dedicated deformation CMR sequences is their time-consuming nature. To overcome this, it is possible to derive strain from feature-tracking of steady-state free precession (SSFP) cine images, with important distinctions being made between 2D (average strain value of three long-axis studies) and 3D derived strain values [42]. Whereas 3D STE is adversely affected by both poor spatial and temporal resolution (leading to coarser speckle patterns) and requires stitching together of volumes to achieve adequate frame rates for analysis at higher heart rates, CMR cine stack datasets are intrinsically three-dimensional with strain quantification highly feasible [42]. Theoretically, 3D strain quantification (either by CMR or STE) overcomes the overestimation of myocardial movement that results from the through-plane loss of features into the third dimension which plagues 2D myocardial deformation techniques [43]. This means that the absolute values of 3D strain are usually lower than that of 2D strain and are likely provide a closer representation of underlying myocardial mechanics [42]. Being relatively novel techniques, the incremental value of CMR feature-tracking derived strain is not well known, with only one study confirming feasibility of 2D CMR feature tracking and its correlation with CMR derived LVEF [44].

**Figure 2:**
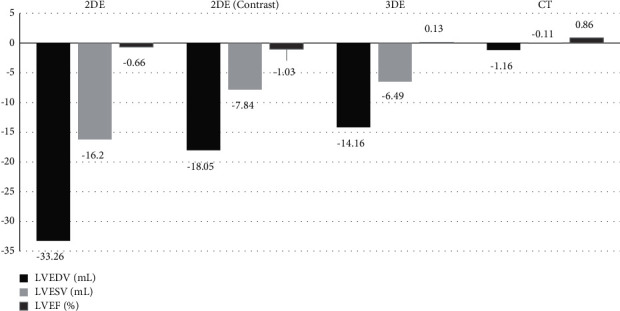
Mean bias associated with LV quantification by multimodality imaging compared to reference CMR using data derived from Rigolli et al. [45].

A large meta-analysis comprising 65 studies and 2888 patients compared the most used noninvasive imaging modalities to the reference standard CMR in the last two decades [45]. The findings revealed significant negative bias in LV end-diastolic volume (LVEDV) and LV end-systolic volume (LVESV) for 2DE ± contrast and 3DE, demonstrating that echocardiography-based techniques tend to underestimate these values, whereas computed tomography (CT) correlates closely, when compared to CMR (Figure 2). In an earlier study involving 114 patients, echocardiography was compared to CMR imaging, focusing on the reference standard for LV function [24]. The study reported that LV volume was consistently underestimated in 2DE and 3DE compared to CMR, and cardiac mass was higher in 2DE than CMR. Compared to CMR, the echocardiographic methods correlated rather poorly, specifically 2D TTE, which demonstrated a low sensitivity (25%) and high false-positive rate (75%) with a mean LVEF 5% higher than CMR. While 3D TTE compared more favourably to CMR and demonstrated less variability, the authors concluded the technique lacks the desired accuracy to detect subtle changes that may have important therapeutic implication.

## 4. Varying Definitions of Cardiotoxicity

Cardiotoxicity is a broad term that refers to any direct untoward toxic effects on cardiac structure and function or the acceleration of cardiovascular disease (CVD) among patients with cardiovascular risk factors or preexisting CVD as a result of cancer therapy [46]. A universal definition for cardiotoxicity is lacking and often oversimplified, resulting in the term being shrouded with controversy due to a lack of clarity. Since the earliest definition of cardiotoxicity was defined [47], definitions used for clinical decisions have varied among different consensus guidelines and clinical trials, usually based on variable cut-off values for LVEF in various imaging modalities [48] (Table 2).

More recently, the European Association of Cardiovascular Imaging (EACVI) and the American Society of Echocardiography (ASE) defined cardiotoxicity as ≥10% decline in LVEF to a final LVEF <53% by echocardiography, multigated acquisition scan (MUGA), and cardiac magnetic resonance imaging (CMR), as well as being the first reported guidelines to include global longitudinal strain (GLS) reduction defined as >15% [16, 19]. The British Society of Echocardiography (BSE) and the British Cardio-Oncology Society (BCOS) have jointly published similar guidelines for adult cancer patients, specifically patients receiving anthracycline ± trastuzumab therapy [17]. The consensus guideline classified cardiotoxicity into three categories: (1) cardiotoxicity, (2) probable subclinical cardiotoxicity, and (3) possible subclinical cardiotoxicity, which should ideally be achieved via advanced echocardiographic measures (2D/3D LVEF and GLS). Additionally, technical considerations should be accounted for due to various factors (clear visualisation endocardial border and timing of measurement during cardiac cycle) that could influence GLS values, thereby further limiting efforts to define abnormal GLS. Establishing a definitive description of cardiotoxicity is vitally important with major clinical implications because while failing to detect cardiotoxicity promptly is harmful, overdiagnosis is equally detrimental, potentially causing interruption to a patient's cancer treatment and thereby impacting upon oncological outcomes.

Trastuzumab has demonstrated effectiveness when used either as monotherapy or in combination with other substances [52]. However, rarely is trastuzumab administered as a single agent, but is instead more commonly combined with surgery, chemotherapy, and radiotherapy as adjuvant therapy. To date, most patients treated with trastuzumab monotherapy have previously been exposed to other forms of treatment such as anthracycline, either prior to, or concurrently with trastuzumab administration. Consequently, the assessment of trastuzumab-related cardiotoxicity is often confounded by the lack of patients with no prior anthracycline exposure. This is important as trastuzumab and anthracycline are considered to have different mechanisms of action. Trastuzumab tends to cause cellular dysfunction in most patients and is perceived to be largely reversible (type 2 cardiotoxicity), whereas anthracycline cardiomyopathy is associated with irreversible myocyte necrosis in the form of apoptosis (type 1 cardiotoxicity) (Table 3) [9]. However, this distinction may be further complicated as recent evidence suggests that trastuzumab could share some common mechanisms with anthracycline-mediated cardiotoxicity, with equally profound toxicity, particularly amongst the elderly population with near-normal ejection fraction and risk factors for CVD. While anthracycline cardiotoxicity is often perceived to be irreversible, there have been reports of partial recovery of cardiac function. Trastuzumab-induced cardiotoxicity is not always reversible [67, 68]. Hence, the classifications of cardiotoxicity so far are oversimplifications, failing to reflect the nuance of its complex pathophysiology and natural history.

## 5. Mechanisms of Trastuzumab Cardiotoxicity

The mechanisms of trastuzumab-induced cardiotoxicity remain to be definitively identified. Limited data from myocardial biopsies reveal rather different mechanisms between trastuzumab and anthracycline, and the prompt recovery of trastuzumab-induced toxicity upon treatment discontinuation further supports this [9]. Different mechanisms have been proposed relating to the cardiotoxic mechanism, while potentially multifactorial and likely attributed to the anti-HER2 activity; this remains a topic for extensive discussion. *In vivo* work in HER2-deleted mice showed interruption to the HER2 signalling pathway resulted in the spontaneous development of dilated cardiomyopathy [75], supporting the notion that HER2 signalling is an important modifier in heart failure. Preclinical studies revealed an overactive HER pathway characterised as overexpression of HER2 receptor on a breast tumour cell or multiple copies of HER2 gene in the nucleus of the cell being the potential underlying mechanism of HER2+ breast cancer [76]. Presently, disruption to NRG/ErbB signalling is recognised as the most likely mechanism of trastuzumab-induced cardiotoxicity. Trastuzumab is known to selectively bind to the juxtamembrane domain IV of HER2 - a section of the extracellular domain essential for HER2 - ErbB4 and HER2-ErbB4 dimerization within the cardiomyocytes. Upon binding, the antibody downregulates the expression of HER2 which initiates a cascade of downstream signalling of the PI3K-AKT-mTOR pathway, which is an important contributor in cellular growth, proliferation, and survival [77]. In patients preexposed to anthracycline, it is probable these patients have begun to undergo subclinical or clinical apoptotic/necrotic process, thereby increasing susceptibility to further myocardial damage. Trastuzumab-associated heart failure is likely the cause of ongoing attrition of myocytes over time.

## 6. Prognosis and Reversibility of Trastuzumab-Induced Cardiotoxicity

In contrast to anthracycline, the clinical outcome for trastuzumab-induced cardiotoxicity is generally considered to be more favourable since LV dysfunction appears largely reversible upon the discontinuation of trastuzumab, and the inclusion of standard cardioprotective therapy seems to accelerate the recovery process [78]. A right ventricular- (RV-) focused CMR study by Barthur et al. [50] found that while RVEF and LVEF had declined with increased RVEDV and RVESV during therapy, all parameters had normalised at 18 months, six months following the cessation of therapy. Consistent with these findings is another study by Ong et al. [72] which utilised feature tracking (FT) strain analysis. The authors reported a reduction in LVEF, FT-GLS, and FT-GCS at 6 and 12 months into therapy. By 18 months, with treatment completed 6 months prior, the parameters returned to near baseline level. Ewer et al. [9] reported on the reversibility of trastuzumab-related LVEF reduction, showing improvements in cardiac function typically at 4 to 6 weeks (before, 0.61 ± 0.13; during, 0.43 ± 0.16; after, 0.56 ± 0.11) following the withdrawal of therapy [79].

Trastuzumab-mediated cardiotoxicity is generally considered to not cause ultrastructural changes, though benign ultrastructural changes were observed from endomyocardial biopsy samples in a trial by Ewer and Ewer [80]. It should be noted that while this is a sensitive method for the evaluation of chemotherapeutic drug-induced cardiotoxicity, its invasive nature and questionable ability to predict clinical outcome renders it impractical for routine clinical use. Moreover, abnormalities uncovered from cardiac biopsy only reflect recent and ongoing changes rather than earlier insults.

**Figure 3:**
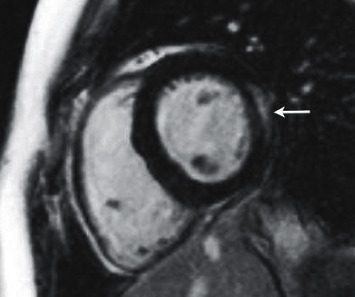
Short axis reconstructed IR-TrueFISP image through the mid-ventricle demonstrates subepicardial linear delayed enhancement (arrow) in the lateral wall of a patient who had received trastuzumab [69]; reprinted with permission from Wadhwa et al. [82].

Additionally, an earlier trial comprising 160 patients by Fallah-Rad et al. [81] identified 10 trastuzumab-induced cardiotoxic patients with subepicardial linear LGE in the lateral portion of the LV. Interestingly, at 6-month follow-up evaluation, despite EF recovery in 6 of the 10 patients, these LGE findings persisted, suggesting persistent myocardial injury. Such findings were amplified in a study by Wadhwa et al. [82] where of the 36 patients that developed mostly asymptomatic cardiotoxicity, subepicardial linear LGE of the LV was observed in 34 patients. Elevation of troponin-I was also reported in 4 patients following >6 cycles of treatment in another trial [83], implying ongoing myocardial necrosis. The underlying mechanism for the presence of LGE is unclear, particularly in the subepicardial lateral portion of the LV, perhaps merely a typical distribution and location associated with this agent (Figure 3).

Relatively little is currently known about the long-term prognosis of trastuzumab-induced cardiotoxicity. To our knowledge, CMR studies to date have seldom followed up patients beyond 18 months. From the available data [50, 72, 73], despite most CMR parameters having demonstrated statistically significant changes at 18 months, the magnitude of reductions is small. This raises the question as to whether these statistically significant reductions are also truly clinically significant for previously cardiotoxic patients or whether they might potentially pose a greater risk of cardiac functional deterioration in the coming years. These findings suggest trastuzumab-mediated cardiotoxicity could be associated with long-term marked impairment of cardiac function and may contribute to increased risk of late-occurring cardiovascular disease in survivors of HER2-positive breast cancer.

One long-term study aimed to determine whether trastuzumab-induced cardiotoxicity recovers and explore any association with long-term cardiopulmonary dysfunction in survivors of HER2+ breast cancer [84]. The trial enrolled 57 patients after completion of trastuzumab-based therapy (median, 7.0 years after therapy). Patients were assessed in three groups using speckle-tracking echocardiography: (1) developed cardiotoxicity during therapy (TOX) group, (2) no evidence of cardiotoxicity during therapy (NTOX) group, and (3) healthy control (HC) group. The study reported significantly lower LVEF in the TOX group (56.9% [5.2%]) compared with the NTOX (62.4% [4.0%]) and HC (65.3% [2.9%]) groups. Similar results were found for GLS (TOX group, −17.8% [2.2%]; NTOX group, −19.8% [2.2%]; HC group, −21.3% [1.8%]) (*P* < 0.001).

A large meta-analysis of randomized and cohort studies of over 29,000 women with breast cancer observed the frequency of severe cardiotoxicity up to 3 years following trastuzumab initiation [85]. Among the 58 studies, severe cardiotoxicity occurred in 844 breast cancer patients, accounting for 3% (95% CI 2.41–3.64) of the total sample. 557 incident cases occurred in the early breast cancer group, 203 in the metastatic breast cancer group, and 84 in the mixed population. Mild or asymptomatic cardiotoxicity was reported in 45 studies with a total incidence case of 2251 (out of 20,491 patients). Two years following the initiation of trastuzumab therapy, severe cardiotoxicity was reported in approximately 3% of the total patient cohort. The incidence rate observed from cohort studies is higher compared to randomized control trials, possibly due to such trials excluding patients at higher risk of adverse events. Accordingly, this renders those studies less reflective of real-world settings. Variability of incident cases between studies was high with frequencies ranging from 0 to 9.8% in the early breast cancer group and 0 to 16.1% in the metastatic group. Such variability of cardiotoxic events is likely associated with patient selection, definition of cardiotoxicity, and methods of assessment.

Based on these findings, the consensus is that trastuzumab-mediated cardiotoxicity is largely reversible, or at least partially reversible, particularly from a functional standpoint. Though the true prevalence and extent of reversibility is debatable, late toxicity remains a possibility. With the toxicity profile of trastuzumab yet to be fully established, treatment necessitates close monitoring, and in the face of new, emerging data, such issues warrant revisiting.

An important limitation to these studies, from the CMR perspective, other than the small sample sizes, is the lack of CMR imaging for evaluating cardiotoxicity, whilst cardiac biomarkers, myocardial biopsy (in some cases), and echocardiography, or other imaging modalities, were adopted instead. Large, prospective CMR-studies are warranted to enable a more definitive conclusion on the diagnostic and prognostic role of trastuzumab-induced cardiotoxicity. Collectively, these studies highlight the potential need for the utilisation of cardiac MRI in the early detection of subclinical cardiotoxicity, as well its extended toxicity profile.

Establishing a validated risk stratification tool to distinguish patients that are at increased risk of developing cardiotoxicity to those of lower risk may be necessary so that monitoring by and utilisation of CMR can be reserved for those at higher risk. From the present data, a multitude of risk factors are associated with increased risk of trastuzumab-treated cardiac events, including age (2.31% in <50 years to 4.91% in >60 years 95% CI 3.22–6.94), postmenopausal status (0.013 OR: 4.39 CI: 1.28–15.02), smoking status (5.3%, 95% CI 2.15–9.75), body mass index ≥25 (6.49%, 95% CI 2.34–12.51), hypertension (5.47%, 95% CI 3.40–7.99), dyslipidaemia (4.43%, 95% CI 2.54–6.78), diabetes (6.19, 95% CI 0.85–15.93), and previous positive cardiac history (19.12%, 95% CI 11.85–27.63) [72, 73]. A scoring system based on these parameters may be valuable for estimating the risk of developing cardiotoxicity during therapy. Additionally, it is important to establish the length of follow-up for previously cardiotoxic patients that are deemed to be at potential higher risk for late toxicity. It is yet to be established if “recovered” patients with mild-to-moderate cardiotoxicity with asymptomatic or oligosymptomatic status possess a higher risk of late toxicity compared to those that developed severe toxicity with intense clinical symptomology.

## 7. Tissue Characterisation and CMR

Chemotherapy associated myocardial oedema, diffuse interstitial fibrosis (collagen deposition in the absence of myocyte loss), and coarse replacement fibrosis (collagen deposition in the presence of myocyte necrosis) can be uniquely imaged with CMR based T2 mapping, T1 mapping, and late-gadolinium enhancement (LGE) sequences, respectively [86] (Table 4). Given the lack of consensus of a precise LVEF-based definition of CTRCD, increasing evidence for deformation imaging to provide incremental prognostic information, and a potential causative relationship between myocardial fibrosis development and reduced myocardial strain [32–34], there is increasing appeal for direct myocardial characterisation in the earlier detection of CTRCD. To date, only the presence of absence of LGE has been studied following trastuzumab therapy [69]. While T1 and ECV increase following anthracycline therapy [87, 88], this has not been characterised for trastuzumab (Table 5).

## 8. Summary: When Should You Do a CMR for Trastuzumab?

**Figure 4:**
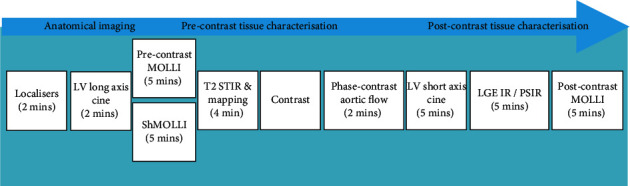
Proposed 30-minute cardiac magnetic resonance imaging protocol for the assessment of trastuzumab cardiotoxicity. LV, left ventricle; MOLLI, modified look-locker inversion recovery; ShMOLLI, shortened modified look-locker inversion recovery; STIR, short tau inversion recovery; IR, inversion recovery; PSIR, phase-sensitive inversion recovery.

In the 2016 ESC position paper, there was recognition of the value of CMR for the following: evaluating cardiac structure and function, identifying the cause of LV dysfunction, and distinguishing left and right ventricular function in difficult cases where other imaging modalities are unsuccessful [1]. Consistent with this are the consensus recommendations from the European Society for Medical Oncology (ESMO) and the joint guidelines from BSE and BCOS, which recommends the utilisation of CMR if significant and unexplained discrepancies exist in echo-derived measures of LVEF and GLS [17, 101]. While CMR is the reference standard procedure for assessing cardiotoxicity, it remains largely underutilised for breast cancer cardiotoxicity surveillance [16]. The choice of imaging modality depends on local expertise and availability; it is strongly encouraged that the imaging modality utilised for baseline assessment remains the same for the remainder of the treatment pathway. A potential protocol for CMR assessment of trastuzumab cardiotoxicity is illustrated in Figure 4.

CMR is demonstrably superior to echo-based imaging of left ventricular function, whether by assessment of LVEF or strain, offering greater sensitivity and specificity in the detection of cardiotoxicity in patients receiving trastuzumab. Furthermore, it offers the ability to assess for myocardial oedema, diffuse interstitial fibrosis, or replacement fibrosis. It also carries some limitations. Thus, currently published normal LVEF reference ranges show an overlap of normal ranges. Application of CMR to patients would require a baseline CMR and regular surveillance scans with associated healthcare costs and requires gadolinium administration.

There are several important lines of enquiry to guide future research. Firstly, whether CMR-based detection of cardiotoxicity as assessed by LVEF and strain leads to improved outcomes compared to their detection by echo remains to be determined. Secondly, whether CMR-based identification of oedema and fibrosis, particularly the type and distribution of the latter, leads to improved risk stratification in trastuzumab cardiotoxicity is unknown. Thirdly, can CMR offer detection of features that would suggest a greater likelihood of recovery from cardiotoxicity by tissue characterisation findings? Given the greater availability of echocardiography than CMR, these three questions are central to further research into the optimum detection, follow-up, and surveillance of cardiotoxicity in trastuzumab patients. In the interim, we agree on the current echo-based methodology of current guidance, with the use of CMR where echo images are poor despite left ventricular opacification with echo contrast agents.

## Figures and Tables

**Table 1:** Advantages and disadvantages of imaging modalities.

Imaging modalities	Cardiac MRI	Echocardiography	Multigated acquisition scan
Advantages	High spatial and temporal resolution	Widespread availability and feasibility	Low inter- and intra-observer variability (<5%)
	Superior signal-to-noise ratio	Low cost	No need for geometric confirmation
	Free choice of imaging plane	Portable	LVEF calculation highly reproducible
	No geometric assumptions required	Current standard and guideline-recommended	

Disadvantages	Expensive	Operator dependence	Repeated exposure to radiation (5–10 millisieverts)
	Lack of portability	Suboptimal acoustic windows	Exposure to radioactive isotope tracers
	Claustrophobic patients unable to tolerate	Use of geometric assumption	Requires venepuncture
	Contraindicated in patients with ferromagnetic metallic implants	High temporal and observer variability	The gamma camera may be suboptimal for critical measurements of EF
	Potential for nephrogenic systemic fibrosis		

**Table 2:** Definitions of cardiotoxicity.

Author and year of publication	Testing modality	Definition of cardiotoxicity	Additional information
Alexander et al. 1979 [47]	Multigated acquisition (MUGA) scan	Mild: decline in LVEF >10%	Anthracycline
		Moderate: decline in LVEF >15% to final LVEF <45%	
		Severe: congestive HF	

Cardiac review and evaluation committee, Seidman et al. 2002 [49]	Echocardiography and MUGA	Drop in global LVEF or more severe in septum	Trastuzumab
		≥5% decline to final EF <55% with symptoms of congestive HF	±
		Asymptomatic decline of ≥10% to final EF <55%	anthracycline

American society of echocardiography (ASE), Plana et al. 2014 [19]	Echocardiography	≥10% decline in LVEF to final LVEF <53%	First guideline to include GLS >15% reduction as definition of cardiotoxicity
		Reduction in global longitudinal strain (GLS) > 15% from baseline	Trastuzumab

Barthur et al. 2017 [50]	Cardiac magnetic resonance	EF < 50%	Trastuzumab

NICE (National Guideline Alliance, 2018) [18]	Echocardiography	LVEF drops by 10 percentage (ejection) points or more from baseline and to below 50%	Chemotherapeutic agents

Keramida et al. 2019 [51]	Echocardiography	≥10% decline in LVEF to final LVEF <50%	GLS reduction >15%
			Trastuzumab

European association of cardiovascular imaging (EACVI), Čelutkienė et al. 2020 [16]	Echocardiography	≥10% decline in LVEF to final LVEF <53%	
		Relative reduction in global longitudinal strain (GLS) reduction by >15% from baseline	

British Society of Echocardiography (BSE) jointly with British Cardio-Oncology Society (BCOS), Dobson et al. 2021 [17]	Echocardiography	The definition is categorised into three groups	Trastuzumab ± anthracycline
		Cardiotoxicity:	
		LVEF: a decline in LVEF by >10 absolute percentage points to a value <50%	
		Probable subclinical cardiotoxicity:	
		LVEF: a decline in LVEF by >10 absolute percentage points to a value ≥50% with an accompanying fall in GLS >15% from baseline	
		Possible subclinical cardiotoxicity:	
		LVEF: a decline in LVEF by <10 absolute percentage points to a value <50%	

Adapted from Lambert, J. and Thavendiranathan, P., 2016. *Controversies in the Definition of Cardiotoxicity: Do We Care? American College of Cardiology*. [online] American College of Cardiology. Available at <https://www.acc.org/latest-in-cardiology/articles/2016/07/07/14/59/controversies-in-the-definition-of-cardiotoxicity> (Accessed 25 August 2021).

**Table 3:** Clinical features differentiating herceptin- and anthracycline-related cardiac dysfunction.

Characteristics	Herceptin (trastuzumab)	Anthracycline (doxorubicin)
Cardiotoxicity	Myocardial dysfunction, also referred to as type II cardiotoxicity	Myocardial damage, also referred to as type I cardiotoxicity
Incidence	2–27%^∗^ [49]	3–26% [53, 54]
Mechanisms	Not definitively understood, though may be multifactorial. The most likely mechanism may be the consequence of attenuated NRG/HER-2 mediated signal transduction pathway and increased susceptibility to anthracycline exposure [55]	Incompletely understood, though may be multifactorial. Potential mechanisms include the following:
		Type IIB topoisomerases-doxorubicin binding [56]
		Disruption of Ca^2+^ homeostasis [57]
		The upregulation of DRs, including TNFR1, Fas, DR4, and DR5 [57]
		Disruption in HER2/HER4 and NRG-1 signalling
		Such mechanisms lead to mitochondrial dysfunction, free radical generation, myocardial oxidative stress, and causing cell apoptosis
Dose effect	Dose-independent [58, 59]	Cumulative, dose-dependent [59, 60]
Features	No ultrastructural changes observed [9, 61]	Ultrastructural changes detected [62]
Clinical course and reversibility	Mostly reversible upon the discontinuation of the agent [9, 63]	Mostly irreversible [64]
Response to cardioprotective therapy	ACEi and *β*-blockers appear to mitigate the risk of HIC [9, 29, 30]	*β*-blockers (particularly carvedilol) have shown promising results in preserving cardiac function. ACEi could mitigate oxidate stress, LV remodelling, and apoptosis. Statins consist of antioxidant and anti-inflammatory properties [65]
		Dexrazoxane may, in part, prevent toxicity by binding to type IIB topoisomerases [66]
Recommencement of agent	Considered relatively safe [9, 46]	High probability of recurrence of dysfunction or cardiotoxicity [9]

ACEi, angiotensin converting enzyme inhibitor; NRG-1, neuregulin-1; HIC, herceptin-induced cardiotoxicity; DRs, death receptors; TNFR1, TNF receptor 1; LV, left ventricular. ^∗^The patient cohort in these trials may have been preexposed to anthracycline.

**Table 4:** CMR findings in herceptin ± anthracycline-treated patients.

	Study design	Size (*N*)	Serial measurement	Definition of cardiotoxicity/CRTCD	Left ventricular function								Right ventricular function			Incidence of cardiotoxicity/CRTCD
					EDV	ESV	EF	MMI	FTGLS	FTGCS	TGLS	TGCS	EDV	ESV	EF	
Fallah-rad et al. [69]	Single centre, prospective	42	12M after treatment initiation	Decline in LVEF of at least 10% below 55%, with accompanying signs or symptoms of CHF	↑	↑	↓	↔								10 (25%)
Grover et al. [70]	Single centre, prospective	15	0, 1, 4, and 12M after treatment initiation	Not reported but significant functional changes characterised as decline in EF of 10%	Mainly ↔ ↑12M	↓	↓						Mainly ↔ ↑12M	↑	↓	Not stated
Nakano et al. [71]	Single centre, prospective	9	0, 3, 6, and 12M after treatment initiation	Cardiac review and evaluation committee criteria			↔3M ↓6&12M									None
Barthur et al. [50]	Multicentre, prospective	41	Three-monthly F/U after treatment initiation for 12M Including an 18m F/U	Not reported			↓6&12M ↔18M						↑6M ↔18M	↑6&12M ↔18M	↓6&12M ↔18M	1, treatment withheld for 1 cycle
Ong et al. [72]	Multicentre, prospective	41	Six-monthly F/U after treatment initiation for 18M	Not reported			↓6&12M ↔18M		↓6&12M ↔18M	↓6&12M ↔18M						1, treatment withheld for 1 cycle
Dhir et al. [73]	Multicentre, prospective	41	Six-monthly F/U after treatment initiation for 18M	LVEF decrease ≥15% from baseline, or LVEF <50% and signs and symptoms of CHF (NYHA class III or IV)	↓6&12M ↔18M	↓6&12M ↔18M	↓6&12M ↔18M									1, treatment withheld for 1 cycle
Houbois et al. [74]	Single centre, prospective	125	Three-monthly F/U after treatment initiation for 12M	Cardiac review and evaluation committee criteria	↑	↑	↓	↑	↓	↓	↓	↓				28% by CMR 22% by 2DE

CRTCD, cancer therapy-related cardiac dysfunction; EDV, end-diastolic volume; ESV, end-systolic volume; EF, ejection fraction; MMI, myocardial mass index; FTGLS, feature tracking global longitudinal strain; FTGCS, feature tracking global circumferential strain; TGLS, tagging global longitudinal strain; TGCS, global circumferential strain; LVEF, left ventricular ejection fraction; F/U, follow-up; M, month; CMR, cardiac magnetic resonance; 2DE, two-dimensional echocardiography; CHF, congestive heart failure; NYHA, New York Heart Association.

**Table 5:** CMR characteristics of chemotherapeutic agents.

	T1	T2	EGE	LGE	ECV	↓ LVEF	↑LV volume	↓ RVEF	Cardiotoxicity/cardiac dysfunction
Herceptin		✓ [89]		✓ [69, 81, 82, 90]		✓ [50]	✓ [73]	✓ [50]	2–27%^∗^ [49]
Anthracycline (doxorubicin)	✓ [87]	✓ [87, 91]	✓ [87]	✓ [90]	✓ [92, 93]	✓ [91]	✓ [70, 94]	✓ [70]	3–26% [53, 54]
Pertuzumab						✓ [56]			6.6% [95]
Lapatinib						✓ [57, 65]			2.7% [96]
Epirubicin			✓ [97]			✓ [97]	✓ [98]	✓ [99]	0.7–11.4% [100]

EGE, early gadolinium enhancement; T1, T1 mapping; T2, T2 mapping; ECV, extracellular volume; LGE, late gadolinium enhancement; LV, left ventricular; ↓ LVEF, reduction in left ventricular ejection fraction; ↓ RVEF, reduction in right ventricular ejection fraction. ^∗^The patient cohort in these trials may have been preexposed to anthracycline.
