# Corrigendum to “Herceptin-Mediated Cardiotoxicity: Assessment by Cardiovascular Magnetic Resonance”

**DOI:** 10.1155/crp/9818679

**Published:** 2025-07-23

J. Jiang, B. Liu, and S. Hothi, “Herceptin-Mediated Cardiotoxicity: Assessment by Cardiovascular Magnetic Resonance,” *Cardiology Research and Practice* (2022), https://doi.org/10.1155/2022/1910841.

In the article, the authors have identified errors in Table 5. The correct Table 5 is shown as follows:

We apologize for this error.

## Figures and Tables

**Table 5:** CMR characteristics of chemotherapeutic agents.

	T1	T2	EGE	LGE	ECV	↓ LVEF	↑ LV volume	↓ RVEF	Cardiotoxicity/cardiac dysfunction
Herceptin		✓ (89)		✓ [69, 81, 82] X (90)		✓ (50)	✓ (73)	✓ (50)	2%–27%^∗^ (49)
Anthracycline (doxorubicin)	✓ (87)	✓ (87, 91)	✓ (87)	X (90)	✓ (92, 93)	✓ (91)	✓ (70, 94)	✓ (70)	3%–26% (53, 54)
Pertuzumab						✓ (56)			6.6% (95)
Lapatinib						✓ (57, 65)			2.7% (96)
Epirubicin			✓ (97)			✓ (97)	✓ (98)	✓ (99)	0.7%–11.4% (100)

*Note:* T1, T1 mapping; T2, T2 mapping; ECV, extracellular volume; ↓ LVEF, reduction in left ventricular ejection fraction; ↓ RVEF, reduction in right ventricular ejection fraction.

Abbreviations: EGE, early gadolinium enhancement; LGE, late gadolinium enhancement; LV, left ventricular.

^∗^The patient cohort in these trials may have been pre-exposed to anthracycline.

